# Severe Spinal Cord Inflammation in a Young Woman Diagnosed With Systemic Lupus Erythematosus: A Case Study

**DOI:** 10.7759/cureus.54325

**Published:** 2024-02-16

**Authors:** Vemparala Priyatha, Aizaz A Shah, Saad Ijaz, Sohaib Ur Rahman, Hidayat Ullah, Wardah Tariq, Noman Salih

**Affiliations:** 1 Internal Medicine, All India Institute of Medical Sciences, Bhubaneswar, Bhubaneswar, IND; 2 Internal Medicine, Hayatabad Medical Complex Peshawar, Peshawar, PAK; 3 Medical-C Unit, Hayatabad Medical Complex Peshawar, Peshawar, PAK; 4 Ophthalmology, Rehman Medical Institute, Haripur, PAK

**Keywords:** plasma exchange, mri spine, nerve conduction study, spinal cord inflammation, longitudinally extensive transverse myelitis, systemic lupus erythematosus

## Abstract

We describe a case of longitudinally extensive transverse myelitis (LETM), an uncommon and dangerous complication of systemic lupus erythematosus (SLE) that struck a 22-year-old woman with SLE. Chronic autoimmune illness (e.g., SLE) affects the skin, kidneys, joints, blood, and neurological system, among other organs. LETM is a condition where the spinal cord becomes inflamed and damaged, causing neurological problems, such as weakness, sensory loss, and bladder dysfunction. The patient presented with abdominal pain, vomiting, body aches, and fatigue, followed by shock, hypoxia, urinary retention, and constipation. Moreover, she had severe and asymmetric weakness, sensory loss, and areflexia in her limbs. She was diagnosed with LETM based on a nerve conduction study and MRI of the spine, which showed a motor neuron disease pattern and T2 hyperintense signals throughout the spinal cord gray and white matter. She responded well to immunoglobulins, plasma exchange, and high-dose steroids as treatment. Although her prognosis is favorable, there might be some lingering neurological issues or limitations. This instance highlights the significance of treating individuals with SLE as soon as possible after developing LETM.

## Introduction

In systemic lupus erythematosus (SLE), the body's tissues and organs are attacked by the immune system. It affects around five million people around the world, but it is more common among Asians and African Americans [1]. The first signs of the disease are usually pain, fatigue, fever, rash, and joint inflammation or pain [1]. The disease can have different effects on different parts of the body, and the symptoms can vary in severity and where they occur.

Myelitis known as lupus myelitis (LM) can strike some patients with SLE, a condition wherein the immune system targets the body's tissues and organs. This can be difficult to detect and treat, occurring in 1%-2% of SLE patients. LM is when the spinal cord is inflamed and damaged, causing nerve problems. LM can be either “complete,” where both legs and the sensations and movements below the spine are affected, or “partial”, where only one leg or some parts of the legs are weak [2]. Acute myelitis is very rare and urgent, affecting only one to four people out of a million. The usual signs and symptoms of acute myelitis are weakness, loss of sensation, and problems with smooth muscles [3]. There is a rare form of myelitis called longitudinally extensive transverse myelitis (LETM), which affects a large part of the spinal cord [4]. Only a few cases of LETM in SLE patients have been reported [5]. Myelitis can be the first sign of SLE in some people, and it can happen even if the SLE is not very active [6]. We report a case of a woman with SLE who had LETM and improved greatly over time with therapy.

## Case presentation

This is a case of a young woman diagnosed five years back as a case of SLE, a chronic autoimmune sickness that affects several organs and can have a rare and fatal side effect. She was diagnosed with SLE as she had antibodies that recognized her nuclear antigens (autoantibodies to nuclear antigens, ANA) and double-stranded DNA (dsDNA) in her blood, both of which are autoimmune indicators. She was taking medications that suppressed her immune system to control her disease, such as azathioprine and hydroxychloroquine. She had been managing well on this therapy, without any major flares or organ damage.

However, she developed abdominal pain, vomiting, body aches, and fatigue, which were preceded by flu-like symptoms, such as fever, cough, and sore throat seven days back. She came to the emergency department with these complaints, and the emergency doctor suspected that she might have a sudden worsening of her SLE, which is called a flare. A flare can inflame and harm different organs, including the kidneys, lungs, heart, brain, and blood vessels. He admitted her to the general medical ward for further evaluation and treatment. He started her on low-dose dexamethasone, a steroid that reduces inflammation, and pulse methylprednisolone, a high-dose steroid given intravenously. He also gave her moxifloxacin as she had signs of possible pneumonia, such as crackles in her lungs and a productive cough.

Despite these interventions, her condition deteriorated, culminating in shock, leading to organ failure. She also had low oxygen levels in her blood and urinary retention. She also had constipation, which is a common side effect of opioids, which she was taking for pain relief. She was transferred to the medical intensive care unit (ICU) for more aggressive care.

In the ICU, the patient was in respiratory distress. She was also hypoxic. She was drowsy and confused, which are signs of altered mental status. Her vital signs were abnormal: her heart rate was 105 beats per minute, her oxygen saturation was 80% on room air, below the typical range of 95-100%, and her blood pressure was a very low 80/50 mmHg. Moreover, she had other symptoms that suggested a problem with her nervous system: she had severe weakness in her legs (she could not move them at all) and uneven weakness in her arms (her right arm was weaker than her left arm). She had lost sensation from her chest down. She did not have any reflexes in her arms or legs, called areflexia.

The patient needed a catheter as she could not urinate on her own. She also needed noninvasive positive pressure ventilation (NIPPV) because she had high levels of carbon dioxide in her blood. Hypercapnia can cause acidosis, which is a condition where the blood becomes too acidic, and it can worsen the patient’s mental status and respiratory function. The ICU team performed many tests to find out the cause of her condition, such as blood tests for D-dimer, complement component 3 (C3), complement component 4 (C4), ESR, and CRP, which are markers of inflammation, clotting, and immune activity; a bedside echocardiogram, which is an ultrasound of the heart; an ultrasound of the abdomen and pelvis; a chest X-ray; and a fluid balance chart, which tracks the input and output of fluids in the body. The ECG showed that she had sinus tachycardia, while the echocardiogram showed reduced left ventricular ejection fraction (40%), which means her heart was not pumping enough blood. The other tests ruled out the possibility of pulmonary embolism, and an acute SLE flare, which means her SLE was not the cause of her condition.

**Figure 1:**
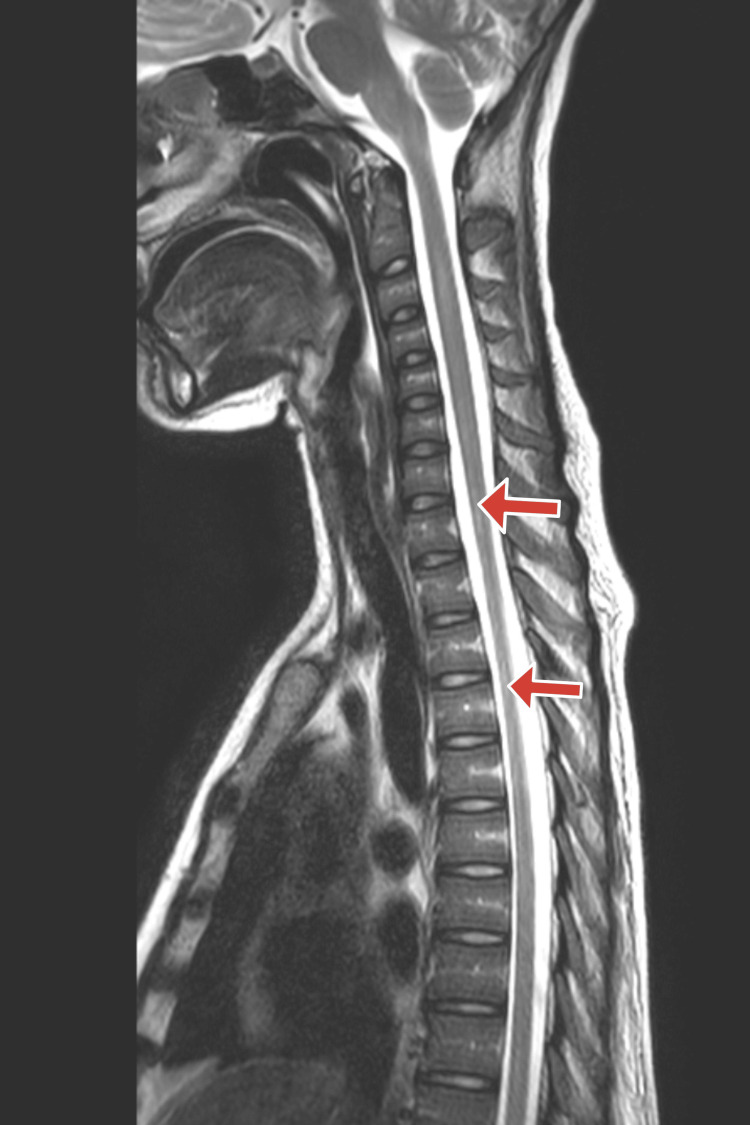
MRI spine with red arrows showing abnormal signals in the spinal cord that were bright on T2 images

**Figure 2:**
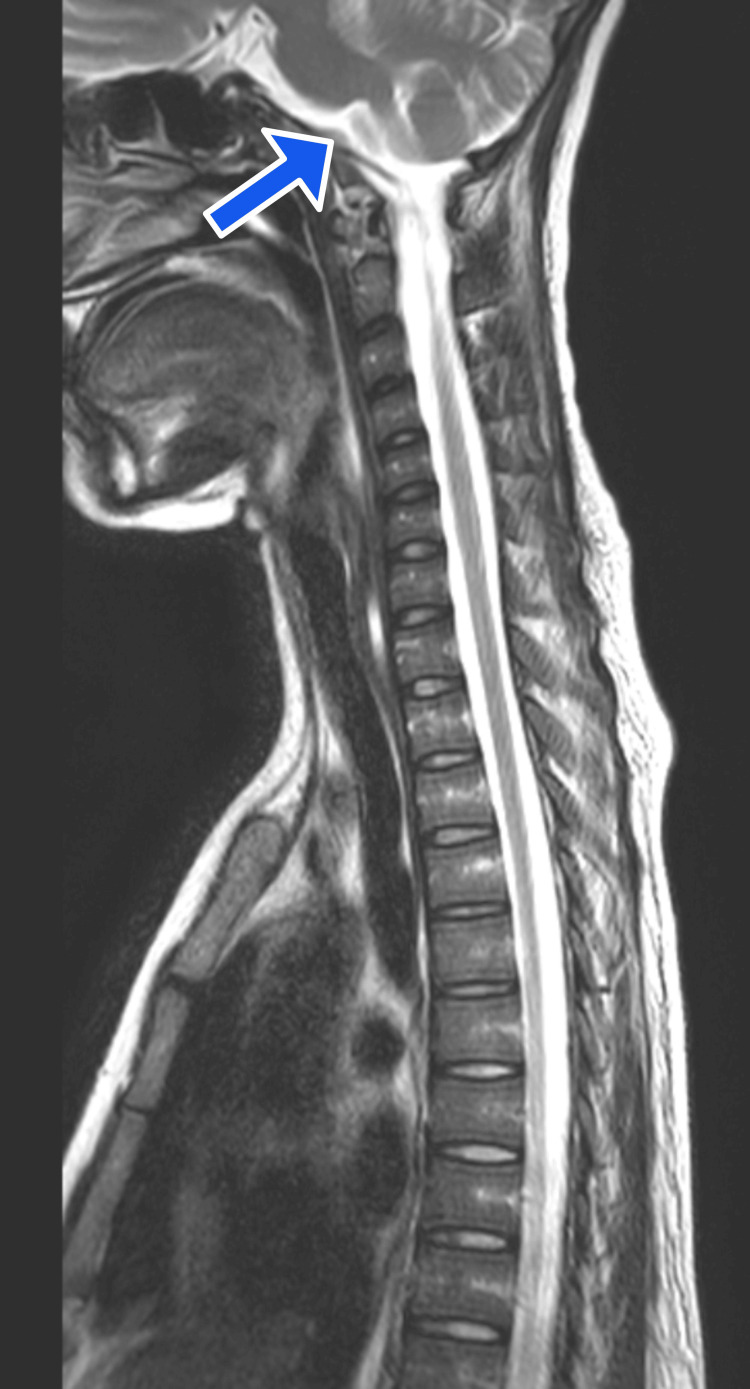
MRI brainstem and spine with a blue arrow showing high signals in the rostral medulla

Based on her history and symptoms, we suspected that she had a rare and serious condition that affects the spinal cord, called LETM. This is when the spinal cord becomes inflamed and damaged, causing neurological problems. We recommended a nerve conduction study (NCS) and an MRI of the spine and brain to confirm the diagnosis. The NCS measures how well the nerves send electrical signals, and it showed a pattern that indicated a motor neuron disease, which is a type of nerve disorder that affects the muscles. The MRI spine showed abnormal signals in the spinal cord that were bright on T2 images, which means they had more water content than normal as shown in Figure 1. These signals involved both the gray and white matter of the spinal cord, which are the parts that contain the nerve cells and the nerve fibers, respectively. The MRI brainstem and spinal cord showed high signals in the rostral medulla with the focus of restricted diffusion on the diffuse weighted image (DWI) shown in Figure 2.

The final diagnosis was LETM, and we started the appropriate treatment, which included high-dose steroids, plasma exchange, and immunoglobulins. These treatments aim to reduce the inflammation and autoantibodies in the spinal cord and to prevent further damage and complications.

The patient is currently showing improvement, with plans to transition from the ICU to the general medical/rehabilitation ward for ongoing observation and follow-up. She will need physiotherapy and occupational therapy to help her regain her strength and function in her limbs. She will also need psychological support and counseling to cope with the emotional impact of her condition. She has a good prognosis, as most patients with LETM recover partially or fully within a year. However, she may have some residual neurological deficits or complications, such as chronic pain, spasticity, bladder dysfunction, or depression. She will need to adapt to her new condition and maintain a good quality of life.

## Discussion

We describe the case of a patient with SLE who also had LETM, an uncommon and dangerous spinal cord disorder. Strong immune-modifying treatment, involving plasmapheresis, cyclophosphamide, and pulse-dose steroids, significantly improved the patient.

The first report of an instance of LETM in an SLE patient was made by Deodhar et al. [7]. Only 22 incidences of LETM in SLE patients were reported in a review of investigations conducted between 1966 and 2008. Although LETM is an uncommon SLE consequence, it can result in varying degrees of impairment. Contrast MRI is the best test to see how bad the disease is [8]. Our patient’s MRI showed that the spinal cord was swollen and bright from C1 to T12. Other causes of LETM can also show similar MRI results, such as infection, MS, and other diseases that affect the immune system, such as Sjögren’s syndrome.

We do not know exactly what causes LETM, but some studies suggest that it might be because of blood clots or inflammation of the blood vessels in the spinal cord, which can lead to a lack of blood and tissue death. It is important to note that the antiphospholipid syndrome, which can cause blood clots, can be linked to transverse myelitis in SLE patients [5]. Our patient did not have any antibodies that were related to the antiphospholipid syndrome or inflammation of the blood vessels.

In people with SLE, there is no conventional therapy for myelitis. According to research, early diagnosis and administration of pulse-dose steroids are critical for the recovery of myelitis in individuals with SLE [9]. Administering cyclophosphamide and pulse-dose steroids combined appears to be more effective than administering pulse-dose steroids alone, according to certain case studies [10,11]. There is also evidence that plasmapheresis, which is a process that removes harmful substances from the blood, can help patients recover faster, especially if they are very sick [12]. Our patient did not get much better after pulse-dose steroids, but she started to improve after plasmapheresis and intravenous cyclophosphamide.

More than one-third of individuals with acute transverse myelitis will recover entirely or virtually completely [13]. Generally, individuals with LETM have a poorer prognosis than those with acute transverse myelitis. Fortunately, our patient with SLE and LETM recovered well over time. As a result, it is critical to detect and aggressively treat LETM as early as possible.

## Conclusions

This case demonstrates an uncommon and dangerous consequence of SLE, LETM. LETM is a disorder wherein the spinal cord gets inflamed and injured, leading to neurological difficulties. The patient presented with abdominal pain, vomiting, body aches, and fatigue, followed by shock, hypoxia, urinary retention, and constipation. Moreover, she had severe and asymmetric weakness, sensory loss, and areflexia in her limbs. She was diagnosed with LETM based on an NCS and MRI of the spine, which showed a motor neuron disease pattern and T2 hyperintense signals throughout the spinal cord gray and white matter. She was treated with high-dose steroids, plasma exchange, and immunoglobulins and showed improvement. She will need ongoing observation and follow-up, as well as physiotherapy, occupational therapy, psychological support, and counseling.

## References

[1] Ishaq M, Nazir L, Riaz A, Kidwai SS, Haroon W, Siddiqi S (2013). Lupus, still a mystery: a comparison of clinical features of Pakistani population living in suburbs of Karachi with other Asian countries. J Pak Med Assoc.

[2] Ford B, Tampieri D, Francis G (1992). Long‐term follow‐up of acute partial transverse myelopathy. Neurology.

[3] Transverse Myelitis Consortium Working Group (2002). Proposed diagnostic criteria and nosology of acute transverse myelitis. Neurology.

[4] Huang LK, Chung CC, Chen BZ, Chi NF, Hu CJ (2013). Systemic lupus erythematosus presented as extensive longitudinal myelitis. Acta Neurol Taiwan.

[5] Espinosa G, Mendizábal A, Mínguez S (2010). Transverse myelitis affecting more than 4 spinal segments associated with systemic lupus erythematosus: clinical, immunological, and radiological characteristics of 22 patients. Semin Arthritis Rheum.

[6] Lehnhardt FG, Impekoven P, Rubbert A, Burghaus L, Neveling M, Heiss WD, Jacobs AH (2004). Recurrent longitudinal myelitis as primary manifestation of SLE. Neurology.

[7] Deodhar AA, Hochenedel T, Bennett RM (1999). Longitudinal involvement of the spinal cord in a patient with lupus related transverse myelitis. J Rheumatol.

[8] Kimura KY, Seino Y, Hirayama Y, Aramaki T, Yamaguchi H, Amano H, Takano T (2002). Systemic lupus erythematosus related transverse myelitis presenting longitudinal involvement of the spinal cord. Intern Med.

[9] Harisdangkul V, Doorenbos D, Subramony SH (1995). Lupus transverse myelopathy: better outcome with early recognition and aggressive high-dose intravenous corticosteroid pulse treatment. J Neurol.

[10] Téllez-Zenteno JF, Remes-Troche JM, Negrete-Pulido RO, Dávila-Maldonado L (2001). Longitudinal myelitis associated with systemic lupus erythematosus: clinical features and magnetic resonance imaging of six cases. Lupus.

[11] Mok CC, Lau CS, Chan EY, Wong RW (1998). Acute transverse myelopathy in systemic lupus erythematosus: clinical presentation, treatment, and outcome. J Rheumatol.

[12] Szczepiorkowski ZM, Winters JL, Bandarenko N (2010). Guidelines on the use of therapeutic apheresis in clinical practice--evidence-based approach from the Apheresis Applications Committee of the American Society for Apheresis. J Clin Apher.

[13] Berman M, Feldman S, Alter M, Zilber N, Kahana E (1981). Acute transverse myelitis: incidence and etiologic considerations. Neurology.

